# Assessment of Cochlear Function during Cochlear Implantation by Extra- and Intracochlear Electrocochleography

**DOI:** 10.3389/fnins.2018.00018

**Published:** 2018-01-26

**Authors:** Adrian Dalbert, Flurin Pfiffner, Marco Hoesli, Kanthaiah Koka, Dorothe Veraguth, Christof Roosli, Alexander Huber

**Affiliations:** ^1^Department of Otorhinolaryngology – Head and Neck Surgery, University of Zurich, University Hospital of Zurich, Zurich, Switzerland; ^2^Department of Research and Technology, Advanced Bionics LLC, Valencia, CA, United States

**Keywords:** cochlear implantation, cochlear implant, electrocochleography, residual hearing, hearing preservation, cochlear trauma

## Abstract

**Objective:** The aims of this study were: (1) To investigate the correlation between electrophysiological changes during cochlear implantation and postoperative hearing loss, and (2) to detect the time points that electrophysiological changes occur during cochlear implantation.

**Material and Methods:** Extra- and intracochlear electrocochleography (ECoG) were used to detect electrophysiological changes during cochlear implantation. Extracochlear ECoG recordings were conducted through a needle electrode placed on the promontory; for intracochlear ECoG recordings, the most apical contact of the cochlear implant (CI) electrode itself was used as the recording electrode. Tone bursts at 250, 500, 750, and 1000 Hz were used as low-frequency acoustic stimuli and clicks as high-frequency acoustic stimuli. Changes of extracochlear ECoG recordings after full insertion of the CI electrode were correlated with pure-tone audiometric findings 4 weeks after surgery.

**Results:** Changes in extracochlear ECoG recordings correlated with postoperative hearing change (*r* = −0.44, *p* = 0.055, *n* = 20). Mean hearing loss in subjects without decrease or loss of extracochlear ECoG signals was 12 dB, compared to a mean hearing loss of 22 dB in subjects with a detectable decrease or a loss of ECoG signals (*p* = 0.0058, *n* = 51). In extracochlear ECoG recordings, a mean increase of the ECoG signal of 4.4 dB occurred after opening the cochlea. If a decrease of ECoG signals occurred during insertion of the CI electrode, the decrease was detectable during the second half of the insertion.

**Conclusion:** ECoG recordings allow detection of electrophysiological changes in the cochlea during cochlear implantation. Decrease of extracochlear ECoG recordings during surgery has a significant correlation with hearing loss 4 weeks after surgery. Trauma to cochlear structures seems to occur during the final phase of the CI electrode insertion. Baseline recordings for extracochlear ECoG recordings should be conducted after opening the cochlea. ECoG responses can be recorded from an intracochlear site using the CI electrode as recording electrode. This technique may prove useful for monitoring cochlear trauma intraoperatively in the future.

## Introduction

Electrocochleography (ECoG) seems to be a promising method to assess cochlear trauma during cochlear implantation. In an animal model, changes in ECoG responses during insertion of an electrode into the cochlea correlated with histological trauma (Adunka et al., 2010; Campbell et al., 2010; Choudhury et al., 2011, 2014; Ahmad et al., 2012; DeMason et al., 2012). The feasibility of ECoG in human cochlear implant (CI) recipients has also been demonstrated (Choudhury et al., 2012; Mandalà et al., 2012; Radeloff et al., 2012; Calloway et al., 2014; Adunka et al., 2015; Campbell et al., 2015, 2016; Dalbert et al., 2015a,b, 2016). Recordings were performed from extracochlear sites (Choudhury et al., 2012; Mandalà et al., 2012; Radeloff et al., 2012; Adunka et al., 2015; Dalbert et al., 2015b, 2016) and from inside the cochlea using either customized recording electrodes (Calloway et al., 2014) or the contacts of the CI electrode itself as recording electrodes (Campbell et al., 2015, 2016; Dalbert et al., 2015a). Almost all human subjects showed some ECoG responses to sound despite substantial levels of hearing loss (Choudhury et al., 2012). Furthermore, some correlation between the assessment of cochlear trauma by ECoG and radiological findings could be demonstrated (Dalbert et al., 2016). However, the predictive value of ECoG changes during cochlear implantation regarding preservation of residual hearing is controversial. Although multiple studies demonstrated a correlation between hearing loss and ECoG changes during surgery for extra- (Mandalà et al., 2012; Radeloff et al., 2012; Dalbert et al., 2015b, 2016) as well as intracochlear recordings (Campbell et al., 2016), contradictory results have also been published (Adunka et al., 2015).

ECoG signals represent electrophysiological responses of the cochlea and the auditory nerve to sound and can provide information about the state of these structures. In CI recipients, these responses are generated by the remaining intact cochlear structures, which are the basis for residual hearing. The ECoG signal combines potentials of cochlear and neural origin. The cochlear microphonic (CM) is a hair cell potential, mainly produced by the outer hair cells. The auditory nerve neurophonic (ANN) and the compound action potential (CAP) are produced by the auditory nerve fibers. The summating potential (SP) most likely has hair cell as well as neural components (Sellick et al., 2003; Forgues et al., 2014).

For the assessment of cochlear trauma during cochlear implantation, the focus of most studies has been on the changes of the CM or the so called ongoing ECoG signal, composed of the CM and the ANN (Radeloff et al., 2012; Calloway et al., 2014; Adunka et al., 2015; Dalbert et al., 2015a,b; Campbell et al., 2016). The CAP has been investigated less extensively (Mandalà et al., 2012; Dalbert et al., 2016). The CM and the ongoing ECoG signal have three distinct advantages over the CAP: (1) Both signals are detectable in almost all CI recipients (Choudhury et al., 2012), (2) Animal studies have demonstrated a better correlation between cochlear trauma and changes of the CM than between cochlear trauma and changes of the CAP (Choudhury et al., 2014), and (3) Both signals show a linear growth up to high-intensity level stimulation (Dalbert et al., 2016). Due to the linear growth, threshold changes and changes of the amplitude near threshold reflect changes at higher intensities. This again allows to record at high intensities where clear ECoG signals are detectable and to avoid time-consuming threshold determinations during surgery.

On the other hand, the correlation between behavioral hearing tests and the amplitude or threshold of the CM or the ongoing ECoG signal is controversial (Campbell et al., 2015; Dalbert et al., 2015a; Koka et al., 2017). Most likely, changes in the CM or the ongoing ECoG signal cannot be directly translated into changes of residual hearing (Campbell et al., 2015; Dalbert et al., 2015a). This could be a reason in favor of using the CAP. It seems reasonable to assume that the purely neural CAP signal has a better correlation to behavioral hearing tests than signals representing hair cell activity, at least in part.

Nevertheless, based on animal studies, a pure hair cell potential would be the best electrophysiological marker to monitor trauma during insertion of an electrode into the cochlea, making the CM a natural choice (Choudhury et al., 2014). However, the often used assumption that the difference of two ECoG signals with alternating starting phases cancels out the neural contribution to the signal and only the CM remains, is not valid at low frequencies and high intensities (Forgues et al., 2014). Consequently, in human CI recipients, a separation of CM and ANN is difficult and to our knowledge, potentials labeled as CM in studies investigating ECoG in human CI recipients cannot be considered as pure hair cell potentials. Thus, the analysis of the ongoing ECoG signal seems to be more adequate as CM and ANN are combined. In this study, we analyzed the ongoing ECoG signal in the low and the CAP in the high frequencies.

This study aimed to accomplish the following: (1) Evaluation of the correlation between changes in extracochlear ECoG recordings at low and high frequencies immediately after insertion of the CI electrode with changes of residual hearing 4 weeks after surgery; (2) Determining electrophysiological changes at different time points during surgery by extra- and intracochlear ECoG.

## Materials and methods

This study is part of a prospective, continuous enrollment study at the University Hospital of Zürich, Switzerland. Part of the data has been previously analyzed and published (Dalbert et al., 2015b, 2016). The study was performed in concordance with the Helsinki Declaration. The study protocol was approved by the Ethical Committee of Zurich (KEK-ZH-Nr. 2013-0317). The indication for cochlear implantation was given after standard evaluations in the CI Clinic of the University Hospital of Zurich, Switzerland. All subjects provided written informed consent before surgery. They were included between November 2013 and December 2016.

All surgeries were performed at the University Hospital of Zurich, Switzerland. A standard anterior mastoidectomy and a maximum size posterior tympanotomy were performed to allow for placement of the extracochlear recording electrode as described later. Then, an anterior-inferior cochleostomy, or an incision of the round window membrane, was conducted. The CI electrode was inserted, and after complete insertion, the site was sealed with soft tissue. Afterwards, the wound was closed in layers and CI telemetry performed. For a detailed description of the surgical procedure we refer to a previous publication (Dalbert et al., 2015b).

Pure-tone testing, performed according to ISO 8235-1, was conducted within 3 months prior to surgery and approximately 4 weeks after surgery. The pure-tone average (PTA) was calculated from the threshold values at 250, 500, 1,000, 2,000, and 4,000 Hz. Hearing loss after surgery was defined as the difference between the pre- and the postsurgical PTA. The maximum output of the audiometer plus 5 dB was used as a threshold value if no response was present at the maximum output of the audiometer.

Statistical analyses were conducted with Stata Statistical Software (Release 13, StataCorp LP, College Station, Texas, U.S.A.).

### Extracochlear ECoG recordings

The Navigator Pro stimulation/recording device and AEP software (Biologic Systems) were used for acoustic stimulation and recording. Before surgery, an insert earphone (Biologic Systems, Mundelein, IL, U.S.A.) was placed in the ear canal for acoustic stimulation. Tone bursts at 250, 500, 750, and 1,000 Hz were used as low-frequency acoustic stimuli, click stimuli as high-frequency acoustic stimuli. Responses to 400 tone bursts or 400 clicks with alternating starting phases were filtered and averaged. The high pass filter was set at 10 Hz, the low pass filter at 3000 Hz for acoustic stimuli at 250, 500, and 750 Hz, at 5,000 Hz for acoustic stimuli at 1,000 Hz, and at 1,500 Hz for acoustic click stimuli. The rise and fall time for tone bursts was 2 cycles shaped by a Blackman window. The plateau phase of tone bursts was 4 cycles at 250 Hz, 10 cycles at 500 Hz, 14 cycles at 750 Hz, and 20 cycles at 1,000 Hz. The recording window was 32 ms for tone bursts and 10.66 ms for click stimuli. The acoustic stimuli were presented at 80–85 dB nHL at 250 Hz, at 85–95 dB nHL at 500 Hz, and at 90–100 dB nHL at 750 and 1,000 Hz. Click stimuli were presented with an intensity of 95 dB nHL.

Standard needle electrodes (20 × 0.3 mm, Neurosign, Magstim Co., Wales, U.K.) were placed in the contralateral pre- or postauricular region (negative), on the forehead (ground), and after complete visualization of the round window on the promontory (positive). Impedances were below 10 kOhm on all electrodes for all ECoG recordings.

Data were exported from the AEP software using the AEP to ASCIII software from Biologic Systems. MATLAB (MathWorks Inc., Natick, MA, U.S.A.) and GraphPad Prism V5.04 (GraphPad Software Inc., La Jolla, CA, U.S.A.) were used for post-processing.

The data from condensation and rarefaction phases were stored separately. The average curve was determined by subtracting both responses and the sum curve by adding both responses. For analysis of the amplitude of the ongoing ECoG signal, the spectrum of each ECoG response was obtained. A time window was defined (9 to 23 ms), isolating the ongoing ECoG signal from the CAP, and a fast Fourier transform (FFT) conducted. The response amplitude at the frequency of the acoustic stimuli (first harmonic) and at the frequency of twice the acoustic stimuli (second harmonic) were determined and added. The sum was defined as the amplitude of the ongoing ECoG signal. An ongoing ECoG signal was considered valid if a response could be visually detected in the average and/or the sum curve and if the amplitude exceeded the mean noise floor plus 3 standard deviations. The mean noise floor and its standard deviation (SD) for each frequency were determined from 173 recordings without acoustic stimulation. To obtain the spectrum of each noise recording, an FFT was performed using the same time window as for all other recordings.

The repeatability of ECoG recordings was assessed by comparing the amplitude of ongoing ECoG signals. Sixty-four ECoG recordings (6 at 250 Hz, 37 at 500 Hz, 16 at 750 Hz, and 5 at 1,000 Hz) were repeated under unchanged conditions before insertion of the CI electrode. The mean amplitude difference was −0.2 dB (SD 0.1 dB).

As in a previous publication (Dalbert et al., 2015a), the sum of the amplitudes of valid ongoing ECoG signals at 250, 500, and 1,000 Hz was defined as the low-frequency ECoG response and taken as a measure of the cochlear function at low frequencies. In concordance with a previous publication (Dalbert et al., 2016), a difference of ≥3 dB between low-frequency ECoG responses was considered relevant. The low-frequency ECoG response was assessed together with the CAP in response to an acoustic click stimulus as high-frequency acoustic stimulus at two time points during surgery: (1) Before opening the cochlea and (2) after full insertion of the CI electrode and sealing of the insertion site with soft tissue. The CAP in response to acoustic click stimuli was assessed visually in the average curve.

In 11 subjects (S59–S62, S65, S66, S68, S69, S72–S74), ECoG recordings were conducted before opening the cochlea, after opening the cochlea, after halfway insertion of the CI electrode, and after full insertion and sealing of the insertion site with soft tissue. For these recordings, one frequency with a clear ECoG response before opening the cochlea was selected and changes of the ongoing ECoG signal at that frequency were analyzed. For the recording, the insertion of the CI electrode was paused and the CI electrode held in an unchanged position by the surgeon.

### Intracochlear ECoG recordings

Intracochlear ECoG recordings were conducted through the HiRes90K CI system (Advanced Bionics, Stafa, Switzerland). The Bionic Ear Data Collection System research software (BEDCS, Advanced Bionics, Stafa, Switzerland) was used. The BEDCS was connected to the CI through the Clarion Programming Interface (CPI, Advanced Bionics, Stafa, Switzerland) and the Platinum Series Speech Processor (Advanced Bionics, Stafa, Switzerland). The amplifier on the HiRes90K CI was configured to have a gain of 1000. The sampling rate was 9,280 Hz. The low pass filter was set at 5,000 Hz. The most apical contact of the HiFocus Mid-Scalar electrode array was used as the recording electrode, the ring electrode as reference electrode.

The acoustic stimulus was generated by a NI DAQ system (NI DAQ 6216, National Instruments Corporation, Austin, TX, U.S.A.) along with an audio amplifier (Sony PHA-2, Sony Corporation, New York, NY, U.S.A.). The sound was presented through ER-3A insert earphones (Etymotic Research Inc., Elk Grove Village, IL, U.S.A.). As acoustic stimulus, a sinusoidal tone burst at 500 Hz with a level of approximately 110 dB SPL was used. The CPI delivered an external trigger to synchronize stimulus generation and ECoG recording through the CI. The recordings were acquired either continuously (S77) or stepwise (S48, S52) during insertion of the CI electrode.

## Results

**Figure 1:**
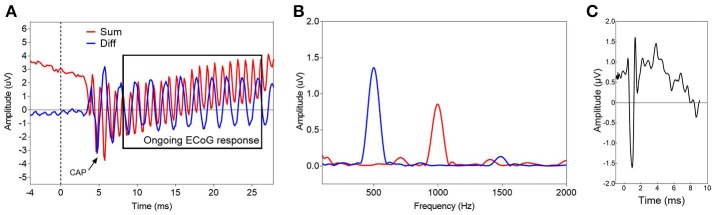
Two examples of typical ECoG responses before insertion of the CI electrode. **(A,B)** show the time waveform **(A)** and the corresponding spectrum **(B)** of an ECoG signal in response to a sinusoidal tone burst with alternating starting phases at 500 Hz, 95 dB nHL (S54). The blue line represents the difference, the red line the average of the responses with alternating polarity. The black rectangle **(A)** marks the time window, used for the spectral analysis. **(C)** Displays an ECoG signal in response to an acoustic click stimulus at 95 dB nHL (S43). Only the average of the responses with alternating starting phases is shown. A clear CAP is visible.

Extracochlear ECoG recordings were conducted in 22 subjects (Figure 1), intracochlear ECoG recordings in 3 subjects (S48, S52, S77). For further analyses, the data was combined with data from 36 additional subjects, which was published previously (Dalbert et al., 2015b, 2016). The demographic, audiometric and electrophysiological data are summarized in Table 1. Subjects included in the two previous publications are marked in Table 1.

**Table 1:** Subject demographics, audiometric, and electrophysiological findings.

**Subject no**.	**Age (Yr)**	**Cochlear implant**	**Round window insertion**	**Preoperative PTA (operated side) (dB HL)**	**Hearing change (operated side) (dB)**	**Hearing change (contralateral side) (dB)**	**Change in low-frequency ECoG response (dB)**	**Change in high-frequency ECoG response**
S1^*^	43	Nucleus CI-512	No	112	Complete HL	2	0.7	–
S3^*^	52	Nucleus CI-422	Yes	70	9	4	−1.3	–
S4^*^	23	Nucleus CI-422	Yes	69	14	−2	−1.8	–
S5^*^	55	Nucleus CI-512	No	101	9	−2	0.6	–
S7^*^	38	HiRes90K HiFocus V	Yes	76	9	1	4.3	–
S8^*^	53	Nucleus CI-422	Yes	76	14	−2	0.6	–
S9^*^	72	HiRes90K HiFocus V	Yes	106	−1	2	−0.4	–
S10^*^	46	HiRes90K HiFocus V	Yes	71	19	No hearing	5	–
S11^*^	46	Nucleus CI-422	Yes	100	10	No hearing	1.9	–
S12^*^	66	HiRes90K HiFocus V	Yes	103	0	1	No response	–
S13^*^	38	HiRes90K HiFocus V	Yes	75	13	3	4	–
S14^*^	23	HiRes90K HiFocus V	Yes	102	11	−5	−1.6	–
S15^*^	64	Nucleus CI-422	Yes	76	Complete HL	−6	−3.8	–
S17^*^	78	Nucleus CI-422	Yes	94	18	1	No response	No response
S18^*^	61	HiRes90K HiFocus V	Yes	82	31	2	1.3	No decrease
S19^*^	59	HiRes90K HiFocus V	Yes	99	8	0	−2.4	–
S21^*^	55	HiRes90K HiFocus V	Yes	112	Complete HL	0	4.5	No response
S22^*^	67	Nucleus CI-422	Yes	101	Complete HL	7	No response	No response
S23^*^	67	Nucleus CI-422	Yes	89	Complete HL	0	−1.9	Loss
S24^*^	60	Nucleus CI-422	Yes	89	Complete HL	0	−0.4	No response
S25^*^	36	Nucleus CI-422	Yes	93	0	No hearing	3.3	Decrease
S26^*^	80	Nucleus CI-512	No	101	10	−7	0.4	No response
S27^*^	61	Nucleus CI-422	Yes	98	3	0	1.7	No decrease
S28^*^	71	Nucleus CI-422	Yes	76	24	3	4.4	No decrease
S29^*^	49	Nucleus CI-422	Yes	78	11	−1	13.5	No decrease
S30^*^	41	Nucleus CI-512	No	96	10	7	1.4	No response
S31^*^	55	Nucleus CI-512	Yes	97	Complete HL	4	No response	No response
S32^*^	30	Nucleus CI-512	No	102	10	1	7.4	No decrease
S34^*^	53	Nucleus CI-512	No	110	5	No hearing	0.9	No decrease
S35^*^	55	Nucleus CI-512	No	98	Complete HL	−1	No response	No response
S36^*^	76	Nucleus CI-512	No	103	15	−12	−6.6	No response
S37^*^	56	Nucleus CI-522	Yes	64	24	−1	3.8	Decrease
S38^*^	38	Nucleus CI-522	Yes	99	4	−1	7.6	No response
S39^*^	42	Nucleus CI-522	Yes	113	4	4	−2.9	No response
S41^*^	53	Nucleus CI-512	No	99	18	2	6.8	No response
S42^*^	53	HiRes90K HiFocus V	Yes	93	22	1	7.3	No decrease
S43	23	Nucleus CI-522	Yes	82	11	1	4.8	No decrease
S44	26	Nucleus CI-512	No	104	14	−1	−7.5	No response
S45	57	Nucleus CI-512	No	99	5	−3	2.5	No decrease
S48	73	HiRes90K HiFocus V	Yes	94	Complete HL	−1		Only intracochlear recordings
S52	74	HiRes90K HiFocus V	Yes	79	10	−8		Only intracochlear recordings
S53	56	Nucleus CI-522	Yes	100	15	7	3.5	No decrease
S54	31	HiRes90K HiFocus V	Yes	74	−1	−5	0.4	No response
S55	64	Nucleus CI-522	Yes	76	12	0	2	No decrease
S58	45	HiRes90K HiFocus V	Yes	103	Complete HL	−1	0.3	No response
S59	61	Nucleus CI-422	Yes	74	24	−7	11.4	No response
S60	60	Nucleus CI-512	No	94	Complete HL	−4	2.1	Loss
S61	43	Nucleus CI-512	No	104	Complete HL	–	−1.3	No decrease
S62	70	Nucleus CI-522	Yes	86	8	−2	2.7	No decrease
S64	55	HiRes90K HiFocus V	No	102	Complete HL	5	−3.2	No response
S65	60	Nucleus CI-522	Yes	64	41	1	0.3	No decrease
S66	29	Nucleus CI-522	Yes	77	9	0	−0.3	Decrease
S67	62	HiRes90K HiFocus V	Yes	102	8	−3	2.3	No decrease
S68	19	Nucleus CI-512	Yes	94	7	−3	−0.9	No decrease
S69	27	Nucleus CI-522	Yes	104	9	−5	3.2	No response
S70	81	HiRes90K HiFocus V	Yes	72	13	0	2	No decrease
S71	72	HiRes90K HiFocus V	Yes	80	31	−4	0.4	Loss
S72	39	Nucleus CI-522	Yes	78	30	−1		Not applicable
S73	52	Nucleus CI-522	Yes	91	−5	0		Not applicable
S74	57	Nucleus CI-512	No	101	3	0	7.8	No decrease
S77	73	HiRes90K HiFocus V	Yes	89	9	0		Only intracochlear recordings

PTA indicates pure-tone average at 250, 500, 1,000, 2,000, and 4,000 Hz; ECoG, electrocochleography; HL, hearing loss;

* *previously published data (Dalbert et al., 2015a, 2016)*.

### Extracochlear ECoG recordings after insertion of the CI electrode and hearing preservation

**Figure 2:**
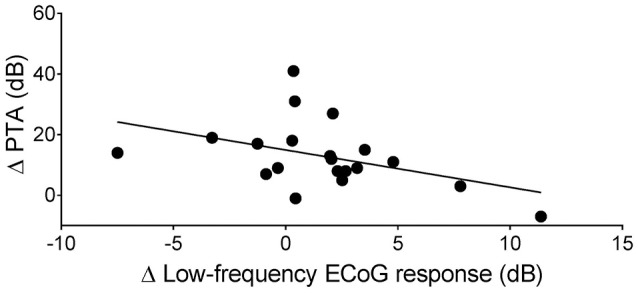
Correlation between the change of the low-frequency ECoG response immediately after full insertion of the CI electrode array (Δ Low-frequency ECoG response) and the change of the pure-tone average 4 weeks after surgery (Δ PTA) (Pearson correlation coefficient, *r* = −0.44, *p* = 0.055, *n* = 20).

In 20 subjects, the low-frequency ECoG response was assessed before opening the cochlea and after full insertion and sealing of the round window with soft tissue. Changes in extracochlear ECoG recordings correlated with the postoperative hearing change (Pearson correlation coefficient, *r* = −0.44, *p* = 0.055, *n* = 20, Figure 2).

**Figure 3:**
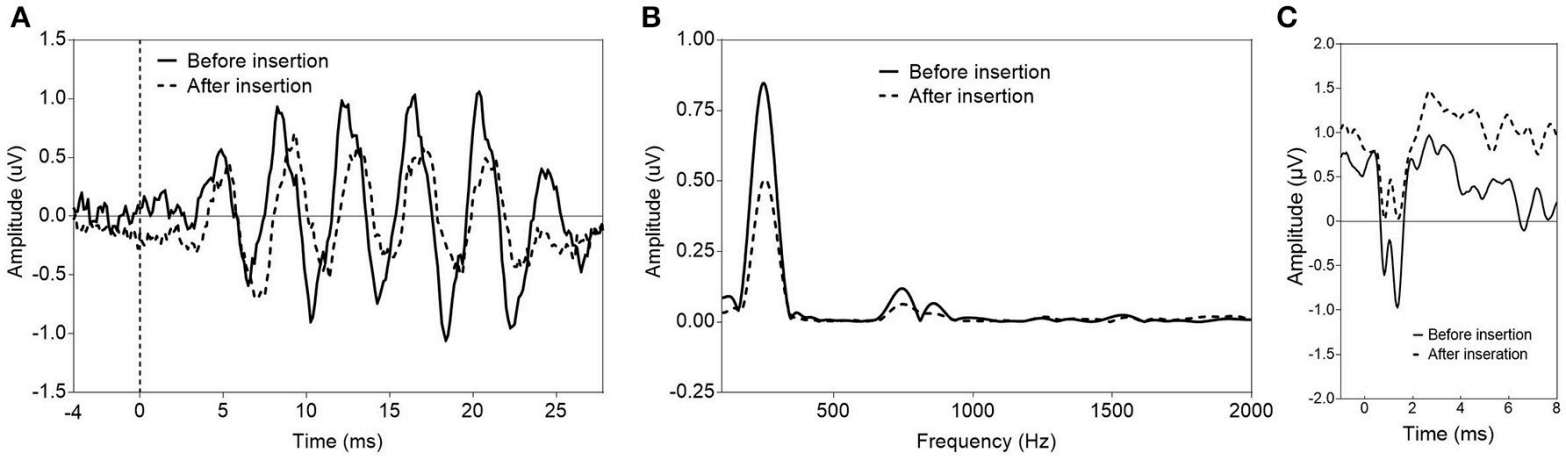
Two examples of a decrease of ECoG signals after insertion of the CI electrode. **(A,B)** show the ECoG response (only the difference curve is shown) in response to a sinusoidal tone burst at 250 Hz, 85 dB nHL before and after insertion of the CI electrode. A decrease of the response amplitude after insertion is visible in the time waveform **(A)** and the corresponding spectrum **(B)** (S64). In S66 **(C)**, a decrease of the CAP amplitude in response to an acoustic click stimulus at 95 dB nHL was detectable after insertion of the CI electrode.

When the data from previous publications (Dalbert et al., 2015b, 2016) was included, a decrease of the low-frequency ECoG response of ≥3 dB occurred in 4/51 subjects (S15, S36, S44, S64) (Figures 3A,B). Subjects with a decrease of ≥3 dB in the low-frequency ECoG response after insertion of the CI electrode had a mean hearing loss of 24 dB at 4 weeks after surgery (SD 14 dB, mean presurgical PTA 94 dB HL); subjects with no relevant decrease in the low-frequency ECoG response, a mean hearing loss of 12 dB (SD 9 dB, mean presurgical PTA 92 dB HL).

A CAP in response to a high-frequency acoustic stimulus was detectable in 16 subjects. Including previously published data (Dalbert et al., 2015b, 2016), a decrease of the amplitude of the CAP or a complete loss of the CAP in response to an acoustic click stimulus after full insertion of the CI electrode was detectable in 6/24 subjects (Figure 3C). This was associated with a mean hearing loss of 21 dB (SD 13 dB, mean presurgical PTA 83 dB HL).

**Figure 4:**
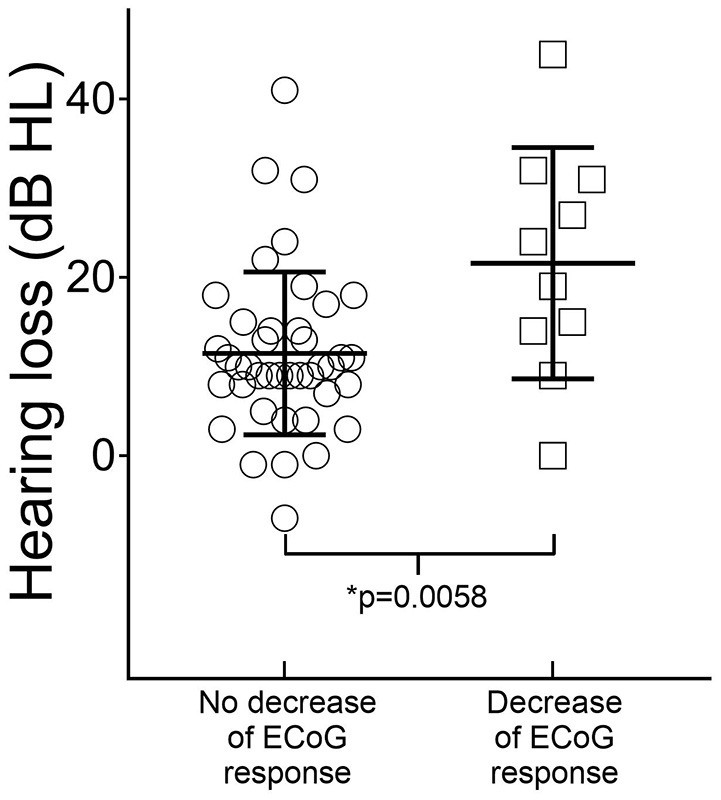
Correlation of hearing loss 4 weeks after surgery with intraoperative ECoG findings. The mean postsurgical hearing loss was 12 dB (standard error of the mean 1.4 dB, *n* = 41) in subjects with no detectable decrease of ECoG signals after insertion of the CI electrode and 22 dB (standard error of the mean 4 dB, *n* = 10) in subjects with decrease of high- or low-frequency ECoG signals (Unpaired *t*-test, *p* = 0.0058).

Overall, in subjects without a decrease or loss of ECoG signals in the high or low frequencies, the mean PTA was 91 dB HL (SD 15 dB) before surgery and 103 dB HL (SD 14 dB) 4 weeks after surgery. In subjects with detectable decrease or loss of ECoG signals, the mean PTA was 87 dB HL (SD 13 dB) before surgery and 109 dB HL (SD 15 dB) after surgery. Therefore, the mean hearing loss in subjects without decrease or loss of ECoG signals was 12 dB, compared to a mean hearing loss of 22 dB in subjects with a detectable decrease or loss of ECoG signals (Unpaired *t*-test, *p* = 0.0058, *n* = 51) (Figure 4).

### Extracochlear ECoG recordings during insertion of CI electrode

**Figure 5:**
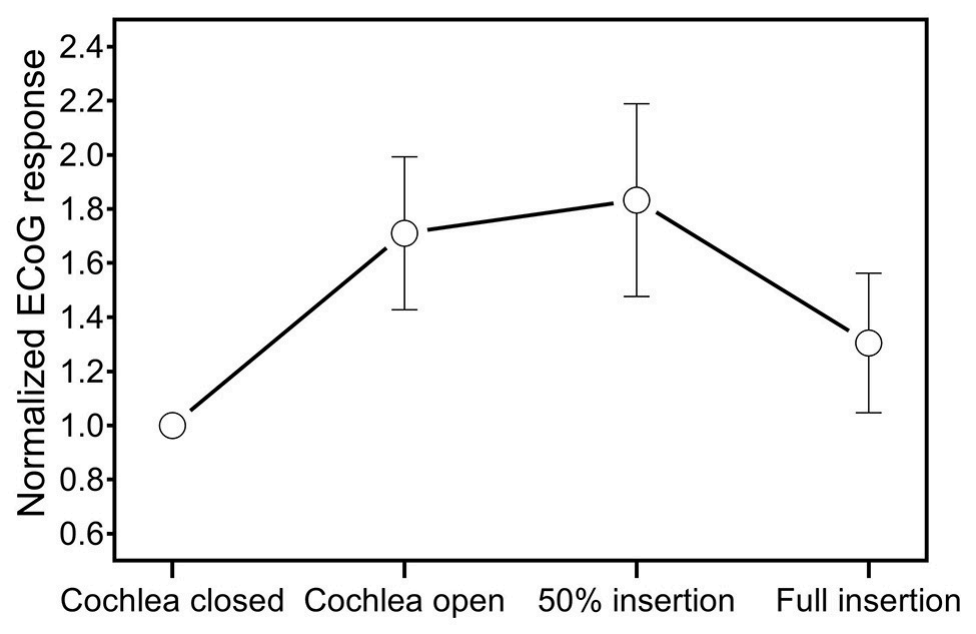
Mean change of the ongoing ECoG signal in extracochlear ECoG recordings during insertion of the CI electrode.

Different patterns occurred in extracochlear ECoG recordings during insertion of the CI electrode (Figure 5). After opening the cochlea, 5/11 subjects (S59, S60, S62, S69, S74) showed an increase of the amplitude of the ongoing ECoG signal of ≥3 dB. Six out of 11 subjects showed unchanged ongoing ECoG responses and no decrease occurred. On average, the amplitude of the ongoing ECoG signal increased by 4.4 dB after opening the cochlea.

During the first half of the insertion of the CI electrode, the ongoing ECoG signals remained unchanged in all subjects. The mean ECoG response amplitude was 29.2 dB re 0.1 uV (SD 6.8 dB) after opening the cochlea and 29.6 dB re 0.1 uV (SD 6.8 dB) after halfway insertion.

During the second half of the insertion, a decrease of the ongoing ECoG signal was detectable in 6/11 subjects (S59, S60, S62, S66, S68, S72). On average, the ECoG response amplitude was 26 dB re 0.1 uV (SD 12 dB) at the end of the insertion. In S72, no valid ECoG signal was detectable after full insertion (amplitude of the ongoing ECoG signal after halfway insertion was 28 dB re 0.1 uV).

### Intracochlear ECoG recordings during insertion of the CI electrode

**Figure 6:**
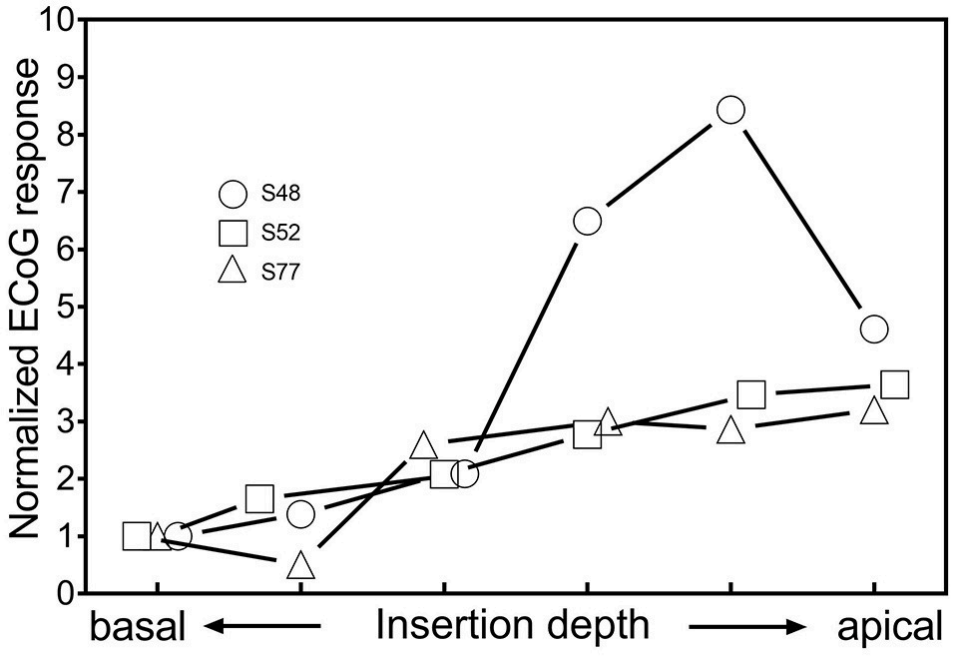
Changes of ECoG signals in intracochlear ECoG recordings during insertion. The most apical contact of the CI electrode itself was used as recording electrode.

The results of the intracochlear ECoG recordings are shown in Figure 6. Two out of 3 subjects (S52, S77) showed an increase of the amplitude of the ECoG signal until the last ECoG recording. In S77, one small, temporary decrease during insertion was detectable, whereas in S52, the ECoG responses continuously increased until full insertion. Subject S48 showed, after an initial increase of the ECoG signal, a decrease of 5.2 dB during the last fifth of the insertion.

## Discussion

As a correlation between histological trauma and a decrease of ECoG responses during insertion of an electrode into the cochlea could be demonstrated in animal studies (Adunka et al., 2010; Campbell et al., 2010; Choudhury et al., 2011, 2014; Ahmad et al., 2012; DeMason et al., 2012), it is plausible to assume that a decrease of ECoG responses in human CI recipients during insertion of the CI electrode represents trauma to cochlear structures. However, although the great potential of ECoG regarding monitoring cochlear trauma during cochlear implantation is generally accepted, the correlation between changes of ECoG signals during surgery and postoperative hearing loss—and therefore the clinical value of such recordings—has still to be proven. Therefore, the aim of this study was to further elucidate the correlation of ECoG changes during surgery and postoperative hearing loss. Furthermore, we aimed to describe at which points during cochlear implantation changes of ECoG signals occur.

### Correlation between changes of extracochlear ECoG responses after insertion of the CI electrode and hearing preservation

Changes in low-frequency ECoG responses correlated with the postoperative hearing change (*r* = −0.44, *p* = 0.055). Subjects with a decrease of high- or low-frequency ECoG signals immediately after insertion of the CI electrode, therefore assumed trauma to cochlear structures during CI surgery, showed a significantly greater hearing loss 4 weeks after surgery compared to subjects without decrease of ECoG signals (22 dB vs. 12 dB, *p* = 0.0058). Subjects with an atraumatic insertion, based on the ECoG recordings, showed a mean hearing loss of 12 dB, corresponding with the amount of hearing loss that is assumed to result from the mechanical changes caused by the insertion of an electrode into the cochlea (Gifford et al., 2008; Gantz et al., 2009; Podskarbi-Fayette et al., 2010). Overall, the presented findings show a significant relationship between trauma during cochlear implantation and loss of residual hearing after surgery. However, a lack of decrease in ECoG signals did not exclude hearing loss exceeding 12 dB or complete loss of residual hearing. This suggests that either postoperative mechanisms independent from cochlear trauma are responsible for postoperative hearing loss or that trauma to cochlear structures occurred but was not detectable by extracochlear ECoG recordings. However, although a decrease of low-frequency ECoG signals seems to be associated with complete or almost complete loss of residual hearing in all cases, a decrease of high-frequency ECoG signals occurred without relevant postoperative hearing loss (S25, S66). In animal studies, changes in ECoG signals were also described when the inserted electrode only touched the basilar membrane but no histologically detectable trauma to cochlear structures resulted (Adunka et al., 2010). Such a mechanism could explain the decrease of high-frequency ECoG responses without relevant postoperative hearing loss.

The addition of high-frequency ECoG recordings, when responses can be detected, increases the information value of ECoG recordings regarding cochlear trauma. A decrease or loss of the high-frequency ECoG response without detectable changes in the low-frequency ECoG response (S23, S25, S37, S60, S66, S71) was associated with a mean hearing loss of 21 dB at 250, 500, and 1,000 Hz and therefore in a majority of cases—except S25 and S66—with a considerable postoperative hearing loss. Had we considered only low-frequency ECoG recordings, these insertions would have been considered atraumatic. Thus far, most studies investigating ECoG changes during cochlear implantation have focused on recordings in the low frequencies (Radeloff et al., 2012; Calloway et al., 2014; Adunka et al., 2015; Dalbert et al., 2015a,b; Campbell et al., 2016). This is an obvious choice, as most CI recipients primarily have low-frequency residual hearing and as hearing preservation is mainly attempted in the low frequencies. However, isolated trauma to high-frequency regions seems to affect hearing preservation in the low frequencies and remains undetected in low-frequency ECoG recordings. We hypothesize that such trauma limited to high-frequency regions triggers postoperative mechanisms that affect low-frequency residual hearing in the postoperative phase.

### Changes of extracochlear ECoG responses during insertion of the CI electrode

The sequential extracochlear ECoG recordings during cochlear implantation showed that the previously described increase of ECoG responses (Adunka et al., 2015; Dalbert et al., 2015a,b, 2016) occurs after opening the cochlea. As discussed in a previous study (Dalbert et al., 2016), intracochlear pressure changes could explain the increase (Ruben et al., 1976). Alternatively, the increase could be caused by contact of the recording electrode with perilymph. As a consequence of this finding, future studies using extracochlear ECoG recordings should conduct baseline recordings after opening the cochlea as a decrease of ECoG signals during the following insertion could otherwise be concealed.

If a decrease of the ongoing ECoG signal occurred during the following insertion of the CI electrode, then the decrease occurred during the second half of the insertion. As for these recordings, acoustic stimuli in the low frequencies were used, two explanations are possible: (1) Cochlear trauma during insertion of the CI electrode occurs mainly during the second half of the insertion and therefore mainly beyond the basal turn, or (2) Cochlear trauma can be detected by low-frequency ECoG recordings only when the CI electrode approaches the tonotopic regions of the acoustic stimulus.

### Comparison of extra- and intracochlear ECoG recordings

Extracochlear ECoG recordings are a reliable tool to assess electrophysiological changes during cochlear implantation. One distinct advantage over intracochlear ECoG recordings is that with the technique described in our study, the placement of the recording electrode remains stable for all recordings. In intracochlear ECoG recordings, the recording electrode moves along the cochlea during insertion, which itself causes a change of the ECoG signal as the relative placement toward the generators of the ECoG signals shifts.

In our study, the number of intracochlear ECoG recordings was not large enough to draw any conclusions. Nonetheless, the findings show the feasibility of this new technique for intraoperative ECoG recordings. Overall, we think intraoperative ECoG recordings using the CI electrode itself as recording electrode hold great promise for the future. The ECoG responses recorded from inside the cochlea are usually much larger and therefore more robust to background noise than extracochlear recordings (Calloway et al., 2014; Dalbert et al., 2015a). Additionally, the sometimes cumbersome placement of an extracochlear recording electrode is circumvented, which facilitates the procedure and makes widespread use in clinical practice more realistic. However, future studies have to investigate the correlation between extra- and intracochlear ECoG findings and thereby allow a more adequate interpretation of intracochlear ECoG recordings.

## Conclusion

ECoG recordings allow for detection of electrophysiological changes in the cochlea during cochlear implantation. A decrease of extracochlear ECoG recordings has a significant correlation with hearing loss 4 weeks after surgery. Therefore, cochlear trauma detectable by extracochlear ECoG recordings seems to be associated with postoperative hearing loss. High-frequency ECoG recordings in addition to low-frequency ECoG recordings add valuable information regarding cochlear trauma. Multiple extracochlear ECoG recordings during surgery revealed a regular increase of ECoG responses after opening the cochlea. Consequently, baseline recordings for extracochlear ECoG recordings should be conducted after opening the cochlea. If a decrease of ECoG responses occurred, the decrease was detectable during the second half of the insertion of the CI electrode. This implies that trauma to cochlear structures occurs toward the end of the insertion of the CI electrode. Intracochlear ECoG recordings seem to be able to detect electrophysiological changes during cochlear implantation but further studies are needed to elucidate the implications of intraoperative findings.

## Ethics statement

This study was carried out in accordance with the recommendations of the Ethical Committee of Zurich with written informed consent from all subjects. All subjects gave written informed consent in accordance with the Declaration of Helsinki. The protocol was approved by the Ethical Committee of Zurich (KEK-ZH-Nr. 2013-0317).

## Author contributions

AD was one of the leading investigators for this study, responsible for study planning, conducting recordings, data post-processing, and was the main author of manuscript. FP was responsible for study planning, conducting recordings, data post-processing, and for reviewing the manuscript. MH was responsible for conducting ECoG recordings and for data postprocessing. KK developed the technique to conduct intracochlear ECoG recordings using the cochlear implant and contributed to writing the manuscript. DV was responsible for study planning and reviewing the manuscript. CR was responsible for study planning, performing CI surgeries, and for contributing to writing the manuscript. AH was the initiator and leader of the study, he also participated in writing and reviewing the manuscript. All authors read and approved the final manuscript.

### Conflict of interest statement

This study was partially funded by Advanced Bionics, Staefa, Switzerland. The authors declare that the research was conducted in the absence of any commercial or financial relationships that could be construed as a potential conflict of interest.
